# A microbial ecosystem: agricultural Jiaosu achieves effective and lasting antifungal activity against *Botrytis cinerea*

**DOI:** 10.1186/s13568-020-01156-7

**Published:** 2020-12-14

**Authors:** Yue Zhang, Youhui Gao, Zehui Zheng, Xingyao Meng, Yafan Cai, Jianbin Liu, Yuegao Hu, Shuangdui Yan, Xiaofen Wang

**Affiliations:** 1 College of Agronomy and Biotechnology, China Agricultural University, Beijing, 100193 China; 2 Beijing Technology and Business University, Beijing, 100048 China; 3 Department of Biochemical Conversion, Deutsches Biomassforschungszentrum gemeinnütziges GmbH, Torgauer Straße 116, 04347 Leipzig, Germany; 4 Institute of Plant Nutrition and Resources, Beijing Academy of Agricultural and Forestry Sciences, Beijing, 100097 China; 5 College of Resource and Environmental Science, Shanxi Agricultural University, Shanxi, 030801 China

**Keywords:** Agricultural Jiaosu, Antifungal activity, Biological agent, Synergistic effects, *Botrytis cinerea*

## Abstract

Synthetic fungicides are eco-unfriendly to agriculture and the environment. Agricultural Jiaosu (AJ), which originates from organic wastes, has the potential to be a substitute for synthetic fungicides. In this study, the characteristics of AJ and its antifungal activity against *Botrytis cinerea* were investigated for the first time. AJ was rich in lactic acid (4.46 g/L), acetic acid (1.52 g/L), *Lactobacillus* (72.45%) and *Acetobacter* (15.23%), which was a microbial ecosystem consisting of acid-based substances (AS) and beneficial microorganisms (BM). The results of the antifungal assays suggested that *B. cinerea* was effectively inhibited by AJ, with the half-maximal inhibitory concentration (IC_50_) of 9.24%. AJ showed the strongest and most-lasting inhibitory effect compared to cell-free supernatant and microbial solution of AJ, indicating that AS and BM and their synergistic effect contributed to the antifungal activity of AJ. Two-step inhibition’ is an antifungal mode of AJ. Firstly, AS not only inhibited the pathogen directly but also provided a dominant niche for BM of AJ. Then, BM in AJ, especially Acetobacter, proliferated and metabolized acetic acid continuously. Thus, AJ achieved high-efficiency and long-acting inhibition. AJ is a promising biological agent considering its features of an eco-friendly, low-cost and easy-to-operate biological agent in rural areas.
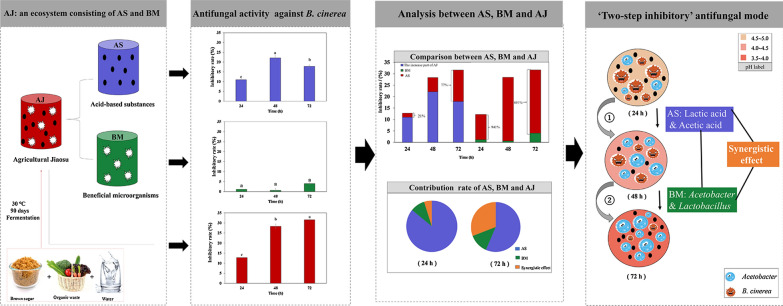

## Key points

1. The antifungal activity of AJ against *B. cinerea* was studied for the first time.
2. AJ was an ecosystem and achieved an effective and lasting antifungal activity.
3. AS, MB and their synergistic effect contributed to higher inhibitory rate.

## Introduction

Phytopathogenic fungi can infect soil and crops, resulting in heavy losses of yield, quality and economy. *Botrytis cinerea* is a common and extremely harmful soil-borne pathogen in agricultural production, which can attack the leaves, flowers and fruits of crops (Santos et al. 2004). Grey mould disease caused by *B. cinerea* occurs on at least 200 plants, especially cash crops, such as vegetables, fruits and medicinal herbs (Panebianco et al. 2015; Xu et al. 2017). *B. cinerea* is a haploid, necrotrophic and heterothallic ascomycete. The complicated aetiology and the multiple ways of aerial growth make it difficult to prevent and control *B. cinerea* (Jeong et al. 2013). At present, several chemical fungicides are used to control *B. cinerea*, including hydroxyanilides, phenylpyrroles, anilinopyrimidines, dicarboximides and carboxamides (Myresiotis et al. 2007). This method is not recommended due to its negative impact on the environment and safety production. More seriously, the long-term application of synthetic fungicides leads to resistance to pathogens and reduces biodiversity (Yildirm and Yapici 2007; Sun et al. 2019; Baraldi et al. 2002). Therefore, it is essential and urgent to develop ecological and sustainable methods to reduce or replace synthetic fungicides (Aqueveque et al. 2017; Rosado-Álvarez et al. 2014).

Currently, substitutes for synthetic fungicides mainly include botanical fungicides and biological agents. Botanical fungicides such as essential oils, chitosan and terpenoids have been reported to have antifungal activity against *B. cinerea* (Aqueveque et al. 2017; Ben-Shalom et al. 2003; Bi and Yu 2016). Biological agents such as *Lactobacillus*, *Saccharomyces cerevisiae* and *Bacillus licheniformis* are effective antagonistic microorganisms (Santos et al. 2004; Trias et al. 2008; Lee et al. 2006). The inhibitory effect of botanical fungicides is stable, but the complex extraction process and high cost make it impossible to apply for all farmers to use. Although biological agents are sustainable, they are not widely used due to cumbersome operation and unstable activity. Thus, high-efficiency, low-cost and eco-friendly products are essential, which should be an ecosystem rich in antifungal substances and antagonistic microorganisms to ensure the inhibitory effect and offset the above shortcomings.

In recent years, Agricultural Jiaosu (AJ) attracts widespread attention and large-scale application as a biological agent in agricultural practice in China. AJ has many advantages, such as sustainability, stabilisation, handleability, low-cost and use of simple equipment. This novel biotechnology can break the bottleneck of fungicides. In general, the AJ is diluted 1:200-500 times and applied to farmland once every 3–7 days. The dosage is about 2–3 L/m^2^. According to the Light Industry Standards of the People’s Republic of China of the Guidelines for Jiaosu Products Classification and Plant Jiaosu, AJ is defined as an ecological product made via fermentation using organic wastes as substrates (MIIT-China 2018a, b). Previous studies have shown that AJ contains acid-based substances (AS), such as lactic acid (LA) and acetic acid (AA), and beneficial microorganisms (BM), such as lactic acid bacteria (LAB) and acetic acid bacteria (AAB); (Selvakumar and Sivashanmugam 2017; Arun and Sivashanmugam 2015; Rahman et al. 2020; Di et al. 2020), which are verified to show the inhibitory ability against *B. cinerea* (Trias et al. 2008; Drysdale and Fleet 1989; Golnaz Hesami 2013; Lagopodi et al. 2009; De Corato et al. 2014; De Vuyst and Leroy 2020). As an ecosystem of AS and BM, AJ has displayed a strong antifungal activity in rural regions of China. However, systematic research is insufficient in the academic community. Scientific experiments are urgently needed to provide theoretical support for the application of AJ.

There is no relevant literature on the antifungal potential of AJ on phytopathogens. In this study, the antifungal activity and mechanism of AJ against *B. cinerea* were investigated for the first time. The research was divided into five aspects: (1) physicochemical properties and microbial characteristics of AJ, (2) changes in the antifungal activity of AJ with dosage, (3) dynamic changes in the inhibitory effect of AJ with cultivation time, (4) main inhibitory factors of AJ and (5) antifungal mode by the synergistic effect between AS and BM of AJ.

## Materials and methods

### AJ and pathogen

Brown sugar (400 g) and jujube wastes (1200 g) were mixed with 4 L distilled water in air-tight containers for anaerobic fermentation at 30 °C for 3 months and stirred once a week. A large number of jujube wastes were produced when farmers harvest at an organic orchards in Jishan County, Shanxi Province, China. Then these wastes were collected to fermentation. The composition of jujube wastes was described by Guo (2019). AJ was centrifuged at 5000 rpm for 15 min. The supernatant was used for further experiments. The volume of the bottle was 5 L with the working volume of 4 L. And mixing the whole fermented system with a stirring rod once a week.

*Botrytis cinerea* (ACCC 36028) was purchased from the Agricultural Culture Collection of China (Beijing, China). The pathogen was cultured on potato dextrose agar (PDA) medium at 25 °C before the experiments.

### Characterisation of AJ

The pH values of AJ were determined by a micro-pH metre (Mettler-Toledo, Greifensee, Switzerland). LA and AA were measured using high-performance liquid chromatography with a diode array detector (SPD-M20A; Shimadzu, Kyoto, Japan). The samples were mixed with acetonitrile at a 1:1 ratio for 10 min and then filtered through a 0.22 μm filter. The details of the chromatographic procedure and conditions were described by Cai et al. (2018a, b).

Man-Rogosa-Sharpe agar was used for the total microbial counts of LAB. AAB counts were determined by a special medium (10 g yeast extract, 10 g dextrose, 20 g CaCO_3_, 0.015 g bromocresol purple, 15 g agar, pH 6.8 ± 0.2). High-throughput sequencing was performed on an MiSeq platform (Personalbio Company, Shanghai, China). The V_3_–V_4_ hypervariable region of the bacteria was amplified using the following universal primer set: 338F (5′-ACTCCTACGGGAGGCAGCA-3′) and 806R (5′-GGACTACHVGGGTWTCTAAT-3′). The community structure was analysed by Silva database at the phylum and gene levels (Zhao et al. 2017). The high-throughput sequencing raw data was deposited in the NCBI Sequence Read Archive database and the accession number was SRR12726966.

### Effect of dosage of AJ on the antifungal activity against *B. cinerea*

Blank control (CK) was prepared without the addition of AJ, whereas 5, 15, 25 and 50 mL AJ were added into 95, 85, 75 and 50 mL autoclaved PDA medium to create the dosage of 5%, 15%, 25% and 50%, respectively, and then shaken gently and poured into the dish. After agar gelling, the mycelial plugs (circle disks) of *B. cinerea* were cut using a 5 mm cork borer sterilised by flame. The plugs were placed at the centre of the plate with different dosages and then incubated at 25 °C for 72 h. The colony diameter was measured every 24 h. All measurements were performed three times. The inhibitory rate was calculated using the following formula (Kuwaki et al. 2002):

$$\eta= \frac{{\left[ {Dck - Di} \right]}}{Di}*100$$

where *η* is the inhibition rate (%), which is indicative of antifungal activity, and *D*_ck_ and *D*_i_ represent the colony diameter of the pathogen of CK and treatments (mm), respectively.

The half-maximal inhibitory concentration (IC_50_) refers to the concentration required for a drug to inhibit cell growth and virus replication by 50%, which is one of the important parameters commonly used in toxicology. Taking the 72 h colony diameter as the *y*-value and the dosage of AJ as the *x*-value, quadratic regression analysis was performed to obtain the fitting curve and polynomial equation. Half of the colony diameter of CK was taken as the *y*-value and entered it into the formula to get the corresponding *x*-value, which is the IC_50_ of AJ against *B. cinerea* (Cai et al. 2018a, b).

### Effect of AJ, cell-free supernatant and microbial solution on the antifungal activity against *B. cinerea*

To reveal the inhibitory factors, an antifungal assay was carried out with the following treatments on AJ: T1, without any treatments; T2, 3 mL AJ was centrifuged at 120,00 rpm for 10 min, and the supernatant was filtered through a 0.22 µm sterile syringe filter (Millex GP, Cork, Ireland) and T3, the precipitate of T2 was washed with sterile water and centrifuged at 12,000 rpm for 10 min, and the supernatant was removed. This operation was repeated three times. Finally, the pellet was mixed with 3 mL sterile water. The same amount of sterile water was used as control (CK). Under the premise of 5% dosage, 19 mL sterile PDA medium was poured into each culture dish. After the medium has cooled and solidified, 1 mL AJ with different treatments was spread onto the surface of fresh PDA medium until drying and then the 5 mm plug was placed at the centre (Kazemipoor et al. 2012) and then incubated at 25 °C for 72 h. The colony diameter was measured every 24 h. Each treatment was repeated three times.

### Dynamic changes in pH, LA and AA during the inhibition process

The above experiment was repeated in PDA medium without agar. The samples were collected at 0, 12, 24, 36, 48, 60 and 72 h to determine the pH value and the concentrations of LA and AA using the methods mentioned above.

### Quantitative polymerase chain reaction (qPCR) analysis of *B. cinerea*, *Lactobacillus and Acetobacter* during the inhibition process

The gene concentrations of *B. cinerea*, *Lactobacillus* and *Acetobacter* were assessed. Sample DNA was obtained at 0, 12, 24, 36, 48, 60 and 72 h during the inhibition process of the above experiments. Real-time qPCR analyses were performed using the ABI fluorescence qPCR instrument (Model 7500; Applied Biosystems, Foster City, CA, USA). PCR consisted of 10 µL using SYBR^®^ Green PCR SuperMix-UDG Kit [Invitrogen, Carlsbad, CA, USA; 0.4 µL Rox dye (10 ×), 0.4 µL primer-F, 0.4 µL primer-R], DNA template (1.0 µL) and double-distilled water to a total volume of 20 µL. The reported units were copies/mL. The PCR primers and the amplification protocol are given in Additional file 1: Tables S1 and S2, respectively.

### Data analysis

Statistical analyses were evaluated based on completely randomised variance at *P* < 0.05 using Statistix 8.1.1 software (Turntech, Beijing, China). Excel 2016 (Microsoft, Inc., Seattle, WA, USA) was used to compute the mean ± standard deviation (SD) and graphical presentation.

## Results

### Characteristics of AJ

To evaluate the antifungal activity of AJ, its characteristics were determined. The main parameters are shown in Table 1. The pH value was 3.48 ± 0.02. The main organic acids were LA and AA, with a concentration of 4.46 ± 0.04 and 1.52 ± 0.02 g/L, respectively. Formic acid (FA), propionic acid (PA) and butyric acid (BA) were not detected. The colony counts of LAB and AAB were 4.78 ± 3.30 and 4.30 ± 2.90 log colony-forming units (CFU)/mL, respectively.

**Table 1:** Characteristics of AJ

Parameter	Value
pH	3.48 ± 0.02
LA (g/L)	4.46 ± 0.04
FA (g/L)	ND
AA (g/L)	1.52 ± 0.02
PA (g/L)	ND
BA (g/L)	ND
LAB (log CFU/mL)	4.78 ± 3.30
AAB (log CFU/mL)	1.30 ± 2.90

*ND* not detected

Mean ± SD (*n* = 3)

**Fig. 1:**
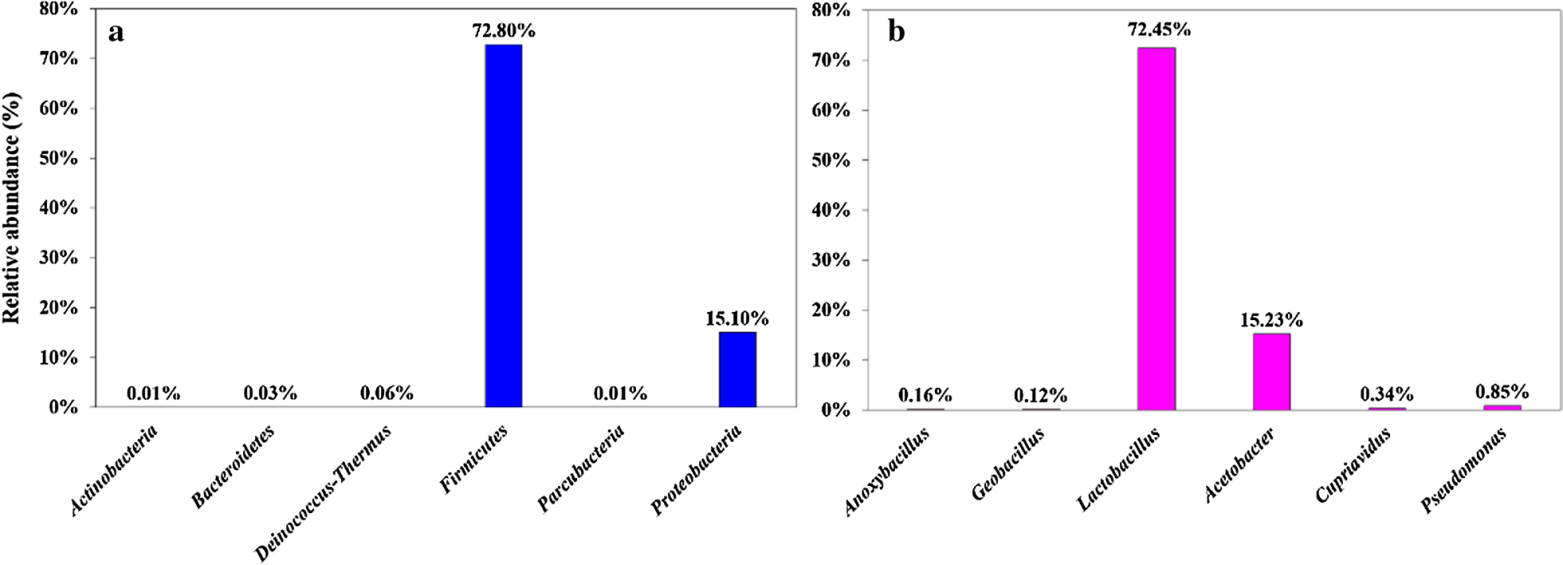
Bacterial communities of AJ: **a** phylum level and **b** genus level

High-throughput analyses were used to detect the bacterial diversity of AJ. Figure 1 shows the bacterial community at the phylum level (a) and genus level (b). *Firmicutes* and *Proteobacteria* dominated the entire community, with relative abundances of 72.80% and 15.10%, respectively. At the genus level, *Lactobacillus* (72.45%) and *Acetobacter* (15.23%) occupied the dominant niche.

AJ was an acidic ecosystem consisting of AS (e.g., LA and AA) and BM (e.g., *Lactobacillus* and *Acetobacter*). It was speculated that AJ had antifungal potential based on its characteristics.

### Effect of dosage of AJ on the antifungal activity against *B. cinerea*

**Fig. 2:**
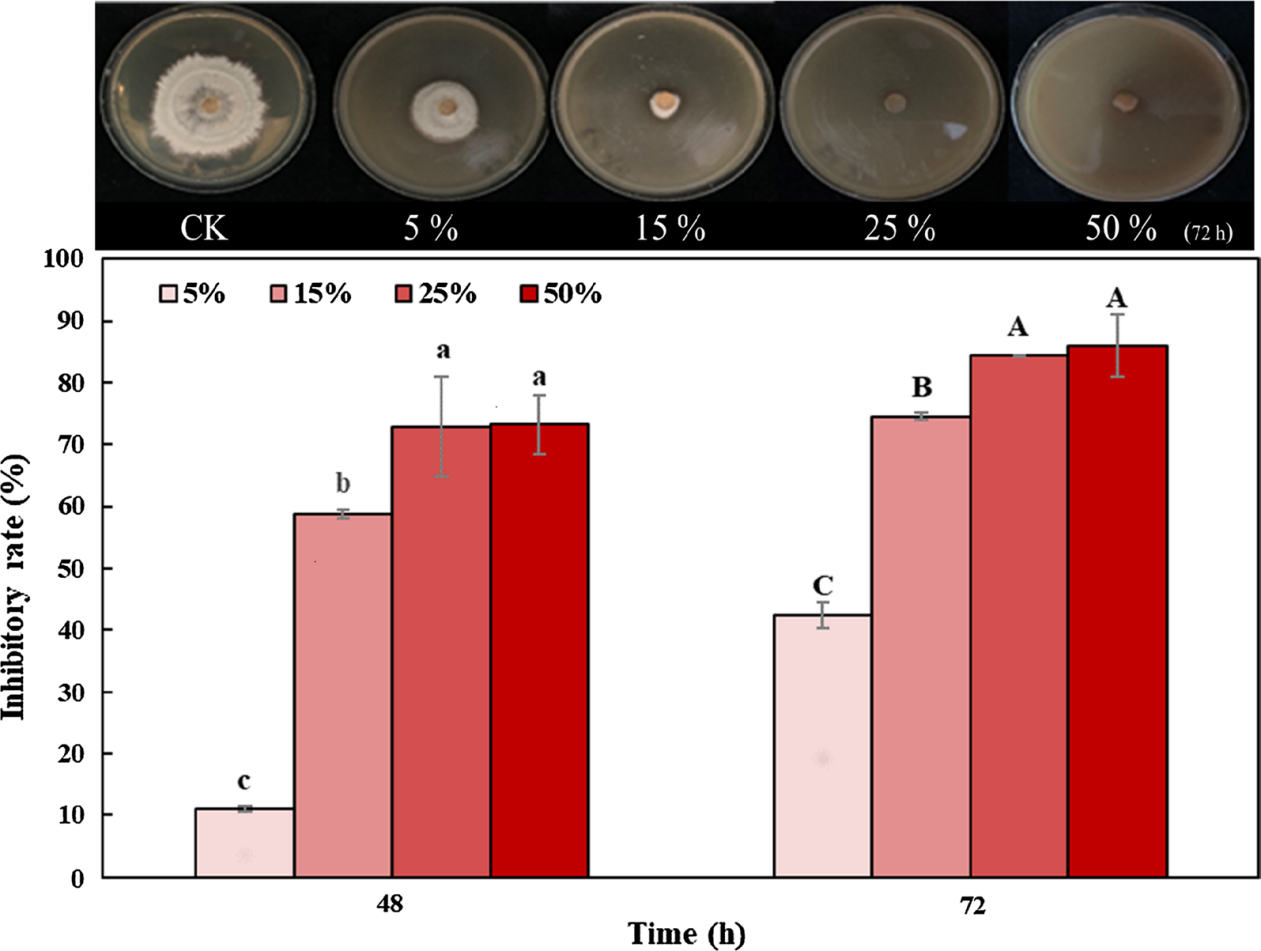
Inhibitory rate at different dosages (photos taken at 72 h)

Dosage is a key factor that affects the antifungal activity in toxicological studies. The effect of the dosage of AJ on its inhibitory effect against *B. cinerea* was investigated (Fig. 2). CK showed the largest colony diameter (52.49 mm) compared to other dosages at 72 h (*P* < 0.05), which indicated that AJ had a negative impact on the growth of *B. cinerea.* The pathogen was almost completely suppressed when the dosage of AJ was more than 15% of the whole medium. Also, the inhibitory rate was positively correlated with dosage and cultivation time. At 48 h, the value of this parameter at dosages of 5%, 15%, 25% and 50% was 10.95%, 58.79%, 74.89% and 73.17%, respectively, but there was no significant difference between the latter two treatments (*P* > 0.05). Interestingly, when the time was prolonged to 72 h, the inhibitory rate climbed to 42.36%, 74.54%, 84.27% and 85.98%, respectively. The inhibition rates of the latter two treatments (25% and 50%) were significantly higher than those of the former two treatments (5% and 15%, *P* < 0.05), but there was no significant difference between them (*P* > 0.05). At this moment, the antifungal activity was 2.87, 0.27, 0.13 and 0.18 times higher than those at 48 h.

IC_50_ was calculated according to the above experimental data using Excel 2016. With the colony diameter on the *y*-axis and the dosage on the *x*-axis, the fitting formula was obtained as follows: *Y* = 0.0382*x*^2^ − 2.7089*x* + 48.021, *R*^2^ = 0.9519. Half of the colony diameter of CK at 72 h (26.245 mm) was introduced into the formula as the *y*-value, and then IC_50_ was taken as the corresponding *x*-value. The IC_50_ of AJ against *B. cinerea* was 9.24% (Additional file 1: Fig. S1), which is of great importance for practical production.

### Antifungal activity of AJ, cell-free supernatant and microbial solution on *B. cinerea*

To determine the main inhibitory factors, AJ was divided into two fractions and the antifungal assay was performed (Table 2). T2 and T3 represented AS and BM of AJ, respectively. T1 was the ecosystem of T2 and T3, which was an ecosystem consisting of AS and BM. The inhibitory effect of each treatment was varied in different periods. At the early stage (24 h), the colony diameter of CK was 25.78 ± 0.45 mm, which was not significantly different from that of T3 (*P* > 0.05) but significantly larger than that of T1 and T2 (*P* < 0.05). At 48 h, the colony diameter of T1 was significantly smaller than that of the other three treatments (*P* < 0.05), but there was no significant difference between T3 and CK (*P* > 0.05). The colony diameter at 72 h of CK, T1, T2 and T3 were 49.27 ± 0.24, 35.78 ± 0.49, 40.29 ± 0.56 and 47.30 ± 0.41 mm, respectively. The difference in treatments was significant (*P* < 0.05). Visually, it was obvious that T1 showed the smallest colony diameter as compared with the other.

**Table 2:** Colony diameter of *B. cinerea* under different treatments

Time (h)	Colony diameter (mm)			
	CK	T1	T2	T3
24	25.78 ± 0.45^a^	22.48 ± 0.40^b^	22.95 ± 0.35^b^	25.48 ± 0.46^a^
48	38.04 ± 0.27^a^	27.65 ± 0.61^c^	29.63 ± 0.68^b^	37.91 ± 0.62^a^
72	49.27 ± 0.24^a^	35.78 ± 0.49^d^	40.29 ± 0.56^c^	47.30 ± 0.41^b^
Photo (72 h)				

CK: blank control using sterile water; T1: AJ, a microbial ecosystem consisting of AS and BM; T2: cell-free supernatant of AJ, only AS; T3: microbial solution of AJ, only BM

Mean ± SD (*n* = 3). Significant differences at *P* < 0.05

**Fig. 3:**
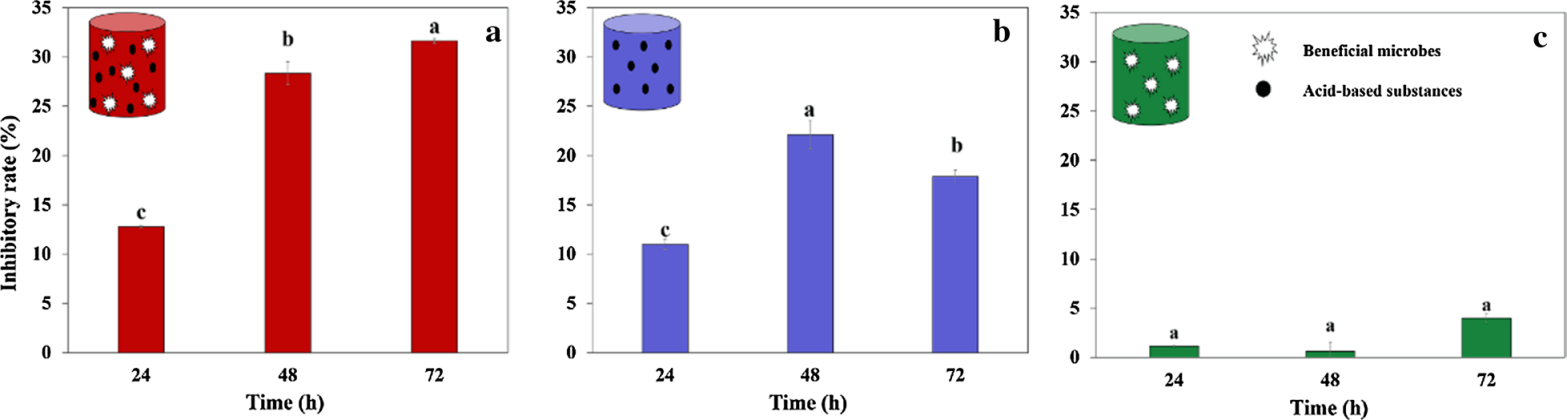
Inhibitory rate of AJ against *B. cinerea:* **a** T1: AJ, a microbial ecosystem consisting of AS and BM; **b** T2: cell-free supernatant of AJ, only AS; **c** T3: microbial solution of AJ, only BM

The difference among the treatments was analysed again from the perspective of the inhibitory rate. T1 exhibited the strongest antifungal activity during cultivation time (Fig. 3a). Its inhibitory rate was 1.16, 1.28 and 1.77 times that of T2 and 10.92, 45.02 and 7.91 times that of T3 at 24, 48 and 72 h, respectively. The inhibitory rate of T1 increased gradually with time, from 12.78% to 31.62% within 72 h (*P* < 0.05). The inhibitory rate of T2 was between T1 and T3 and the value increased at the beginning and then decreased (*P* < 0.05). The peak appeared at 48 h, with 22.11% (Fig. 3b). The inhibitory rate of T3 was low, but it showed an increasing trend. The inhibition rate increased from 1.17% to 4.00% during the inhibition process (Fig. 3c).

In general, the antifungal activity AJ was the most outstanding, with gradual stronger inhibition. The inhibitory rate of cell-free supernatant was lower than that of AJ at the early stage, and this value decreased remarkably at the late stage. Microbial solution had weak inhibition against the pathogen but showed an increasing tendency.

### Dynamic changes in pH, LA and AA during the inhibition process

**Fig. 4:**
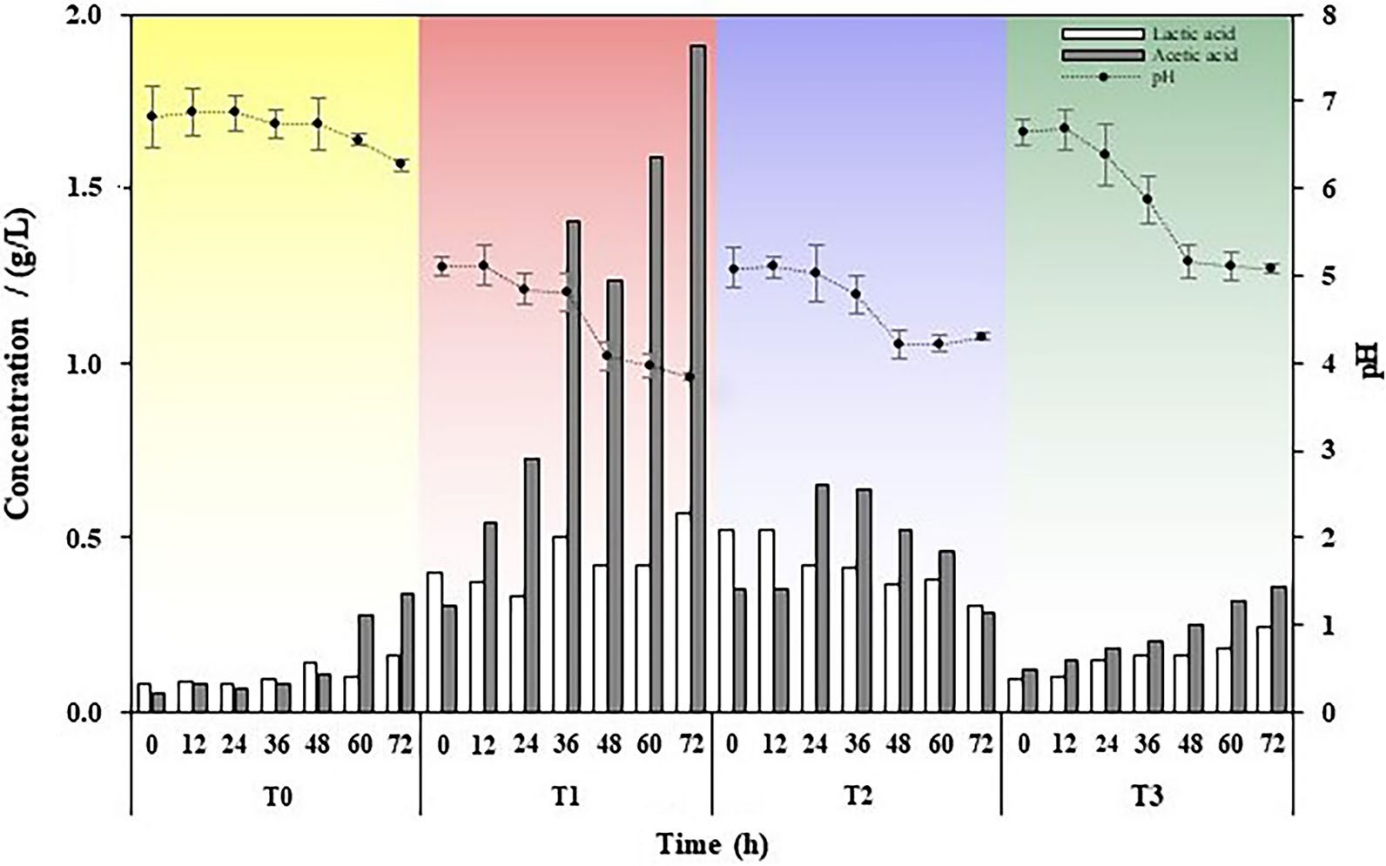
Dynamic changes in the pH value and concentrations of LA and AA

To further reveal the antifungal mechanism of AJ, the concentrations of LA and AA and the pH were dynamically measured during the inhibition process (Fig. 4). Organic acids of T1 gradually accumulated, especially AA, whose concentration increased from 0.31 to 1.91 g/L. With the addition of AS of AJ, the initial pH value was only 5.10 and then reduced to 3.84 (*P* < 0.05). The concentration of LA showed a decline with cultivation time from 0.52 to 0.31 g/L. As for AA, there was a trend of increase first and then gradually decreased to 0.29 g/L. Finally, the concentration of AA was only 15.18% of T1. The main component of cell-free supernatant was AS, so the pH value was only 5.09 at the beginning. Subsequently, the value dropped temporarily and then increased. The lowest pH value was observed at 48 h (4.21). Microbial solution consisted of BM of AJ, such as *Lactobacillus* and *Acetobacter*. Compared to T1, LA and AA in T3 increased slightly. The concentration of AA was only 0.36 g/L at 72 h, which was 18.85% that of T1. The pH value dropped from 6.66 to 5.08, which was equivalent to the initial value of T1.

In summary, the pH value and the concentration of AA displayed an apparent fluctuation during the inhibition process. AJ showed the lowest pH value and the strongest acid production capacity, especially AA.

### qPCR analysis of *B. cinerea*, *Lactobacillus and Acetobacter* during the inhibition process

**Fig. 5:**
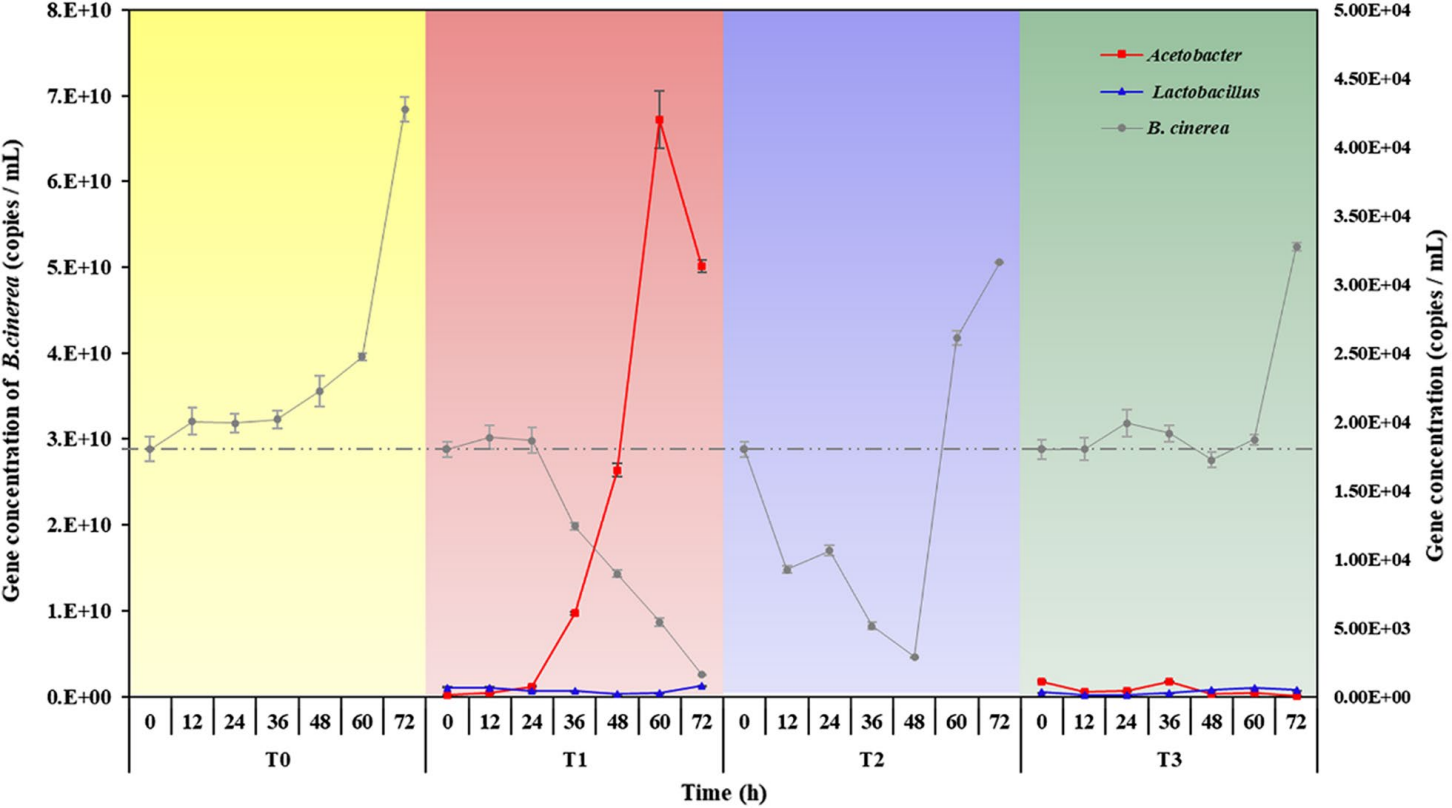
Gene concentrations of *B. cinerea*, *Lactobacillus* and Acetobacter under different treatments (CK: blank control using sterile water; T1: AJ, a microbial ecosystem consisting of AS and BM; T2: cell-free supernatant of AJ, only AS; T3: microbial solution of AJ, only BM), and the label at the left and right Y-axis represents gene concentration of *B. cinerea* and bacterial, respectively

*B. cinerea* and the main strains of AJ (*Lactobacillus* and *Acetobacter*) in different periods were quantitated by qPCR (Fig. 5). *B. cinerea* of CK grew continuously, with gene concentrations from 1.80E+04 to 4.27E+04 copies/mL. In contrast, the pathogen was inhibited effectively during the inhibition process in T1, and the gene concentration decreased to 1.61E+03 copies/mL. Simultaneously, *Acetobacter* in AJ increased exponentially and rapidly became the dominant microorganism, whose gene concentration reached a peak at 60 h (6.72E + 10 copies/mL) from 1.68E+08 copies/mL at 0 h. Cell-free supernatant (T2) showed an inhibitory effect at the early stage. The gene concentration of the pathogen decreased to 2.89E+03 copies/mL within 48 h and then recovered to 3.16E + 04 copies/mL. Microbial solution (T3) had a weak influence on the growth of *B. cinerea*, with the highest gene concentration of 3.27E + 04 copies/mL at 72 h.

In summary, AJ could inhibit the pathogen effectively and persistently. *Acetobacter* rapidly proliferated and occupied the dominant niche, thus inhibiting the growth of the pathogen by metabolising a large amount of AA. This conclusion was consistent with the results of Fig. 4.

### Contribution rates of AS and BM of AJ and their synergistic effect

In Fig. 3, the final antifungal activity of AJ (T1) was better than that of cell-free supernatant (T2) and microbial solution (T3). Additional file 1: Fig. S2 was obtained by comparing T2 and T3 to T1. The red area in the columns represents the increase part of the inhibitory rate of AJ compared to that of the cell-free supernatant and microbial solution. At the early stage (24 h) and late stage (72 h), AJ increased the inhibitory rate by 21% and 77% compared to cell-free supernatant and 9.41 and 6.91 times compared to microbial solution. These results showed that AJ, as an ecosystem of AS and BM, had a stronger inhibitory effect against *B. cinerea*.

**Fig. 6:**
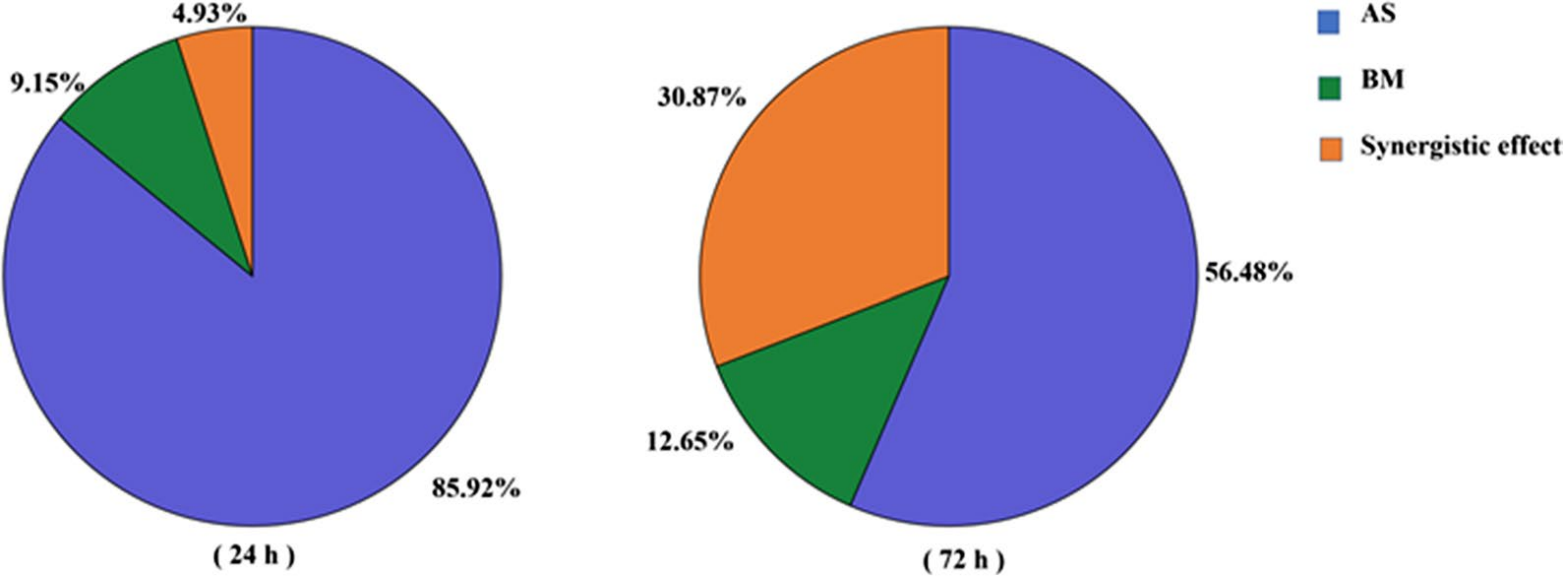
Contribution rate of AS and BM and their synergistic effect

When the inhibitory rates of T2 and T3 were added, the sum was less than the inhibitory rate of T1. This difference was considered as a synergistic effect of AS and BM. Figure 6 shows the percentage of the contribution rate of AS and BM and their synergistic effect at the beginning and end. AS accounted for the largest proportion at 24 h and then declined from 85.92% to 56.48%. Conversely, the contribution rate of BM increased gradually with prolonged cultivation time, with the value increasing from 9.15% to 12.65%. Likewise, the proportion of synergistic effects also had a sharp increase, which reached 30.78% at the late stage (72 h).

In summary, AS were the main inhibitory factor at the early stage. BM gradually played a vital role at the late stage, which led to an increased proportion of the synergistic.

## Discussion

AJ not only has a strong antifungal activity but also provides a high-value-added treatment strategy of organic wastes, which achieves the dual role of resource utilisation and safety production. This product has the advantages of green, low-cost and easy operation and has been widely applied in rural China in recent years (Zhang et al. 2020). In this study, the antifungal activity and mechanism of AJ on *B. cinerea* were investigated. The following points were discussed based on the comprehensive analysis of the results.

AS and BM and their synergistic effect contributed to the antifungal activity of AJ against *B. cinerea*. AJ is a fermentation ecosystem that integrates AS (LA and AA) and BM (*Lactobacillus* and *Acetobacter*). When AS and BM of AJ were separated, the two fractions still showed an inhibitory effect. These results suggested that AS and BM are the key inhibitory factors. *Acetobacter* was the antagonistic microorganism that played a major role during the late stage of the inhibition process (Fig. 5). Also, the inhibitory effect of AJ was more favourable than that of cell-free supernatant and microbial solution, which indicated that the synergistic effect between AS and BM is another factor that could not be ignored.

Figure 2 shows that the antifungal activity of AJ was positively correlated with dosage. The production of AJ is a fermentation process dominated by BM in which sugar is converted into organic acids and the acids gradually accumulate (Arun and Sivashanmugam 2015; Selvakumar and Sivashanmugam 2018). Our results also proved that AJ was an ecosystem that contains AS, mainly LA and AA (Table 1). The organic acids can change the permeability of the cell membrane of the pathogen, make antifungal components such as AA enter the target pathogen (Hashemi et al. 2017), reduce the cytoplasmic pH, suspend cell metabolism and directly inhibit the growth of the pathogen (Dalié et al. 2010; Yamaji et al. 2005). The inhibitory rate of AJ increased with dosage, which may be related to the concentrations of organic acids. When AJ was added to PDA medium at the ratio of 5%, the concentration of LA and AA was 0.23 and 0.08 g/L, respectively. The concentration of the two organic acids increased by 10 times when the ratio was 50%.

The antifungal activity of AJ changed with cultivation time (Figs. 2 and 3; Table 2). AJ (an ecosystem of AS and BM) had the strongest inhibitory effect and became gradually stronger. Cell-free supernatant (only AS) showed slightly lower performance than AJ at the early stage, and the inhibition rate was significantly reduced at the late stage. The antifungal activity of microbial solution (only BM) was weak, but it showed a slightly stronger characteristic after 48 h. In Fig. 2, the inhibitory rate of the three treatments at 72 h was higher than at 24 h, which also proved that the inhibitory effect of AJ gradually stronger. Moreover, Fig. 3 also shows that the antifungal activity of AJ increased with cultivation time. AS in AJ inhibited the pathogen effectively in a short time and BM of AJ, especially *Acetobacter,* occupied the niche simultaneously.

The inhibitory effect of cell-free supernatant increased at first and then decreased (Fig. 3b). The qPCR results revealed that the genes of *Lactobacillus* and *Acetobacter* were not detected in T2, which indicated that the microorganisms of AJ had been removed (Kazemipoor et al. 2012; Li et al. 2013), and AS performed antifungal activity mainly at this time (Chen et al. 2007; Stoianova et al. 2012). AS have a positive impact on antifungal activity (Golnaz Hesami 2013; De Corato et al. 2014). However, previous studies have shown that organic acids can be consumed or transformed by microorganisms as carbon sources during natural fermentation (Rao et al. 2020; Zacharof and Lovitt 2012; Huang et al. 2016; Liu et al. 2008; Baskaran et al. 2002); thus, the antifungal activity became weak. Figure 4 also illustrated this conclusion in which the concentration of LA and AA decreased in T2 during the inhibition process. This result was similar to the results of Liu et al. (2006); when the concentration of AA in fermented broth was reduced from 4.27 to 4.14 g/L and that of LA was decreased from 17.45 to 17.32 g/L, the diameter of the bacteriostatic zone declined from 36 to 26 mm.

Compared to cell-free supernatant and AJ, the antifungal activity of microbial solution increased slightly with cultivation time. Probiotics often coexist with harmful bacteria and play a beneficial role only when they occupy the niche (Oh et al. 2017). Figure 5 shows that the gene concentration of *Lactobacillus* and *Acetobacter* was extremely low during the inhibition process. Therefore, BM of AJ failed to become the dominant bacteria by overcoming the vigorous growth of *B. cinerea*. However, *Lactobacillus* and *Acetobacter* were active and metabolised a small amount of organic acids (Fig. 4). Then, the pH value decreased slightly and the inhibitory rate increased at the late stage.

There was a synergistic effect between AS and BM, which led AJ to have a ‘1 + 1 > 2’ inhibitory effect. Figure 6 shows that the antifungal activity of AS became weaker and weaker, whereas the contribution rate of BM increased gradually. This is because the inhibitory effect of cell-free supernatant was weakened by a decreased concentration of AS. Similar studies have been reported. The antibacterial effect of the supernatant of LAB fermentation became weaker with the decrease of LA and AA (Li et al. 2008; Li et al. 2006). However, *Acetobacter* rapidly propagated and continuously produced AA, which indirectly affected the growth of *B. cinerea* (Lefeber et al. 2011; Myresiotis et al. 2007). Meanwhile, the contribution rate of the synergistic effect also increased by 5.62 times. This was the similar with the results of Tadijan et al. (2016), the inhibitory effect of antagonistic microorganisms on the phytopathogenic fungus was gradually enhanced within 72 h.

**Fig. 7:**
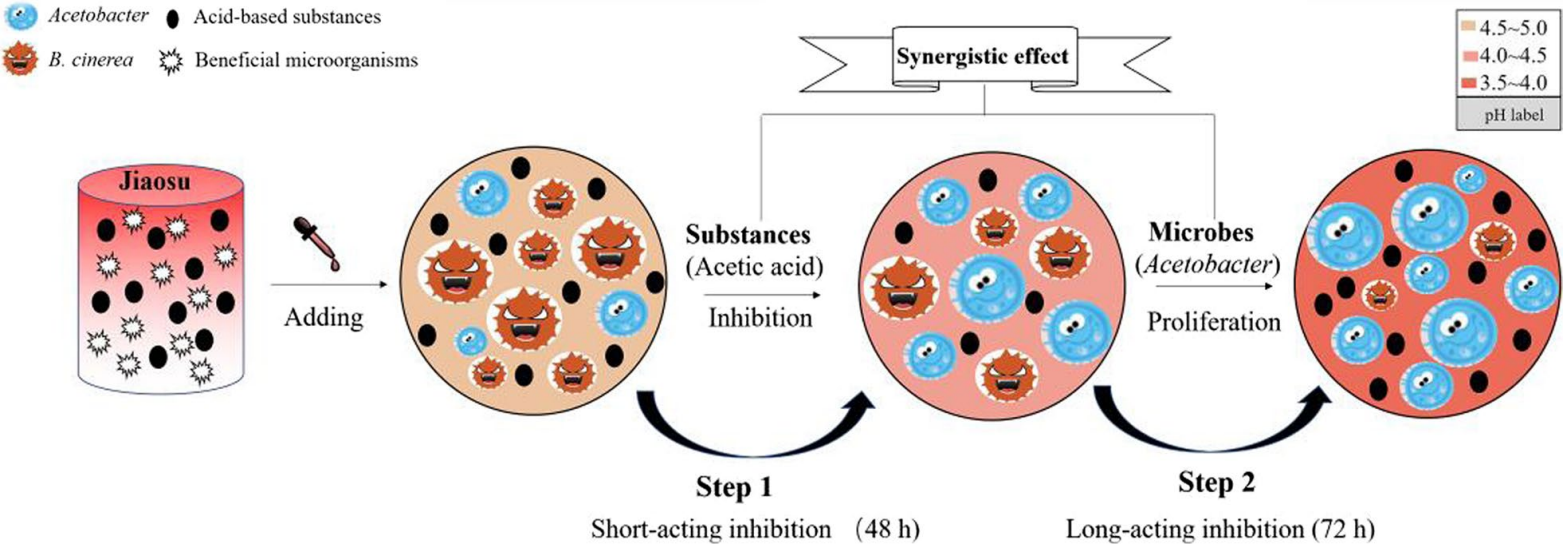
‘Two-step inhibition’ antifungal mode of AJ

Based on the above analysis, an antifungal mode called ‘two-step inhibition’ of AJ was proposed, as shown in Fig. 7. AJ was rich in AS (AA and LA) and BM (*Acetobacter* and *Lactobacillus*). When AJ was added to the medium dominated by phytopathogens, AS inhibited the growth of the pathogen directly at the early stage (48 h) and provided a dominant niche for BM in AJ simultaneously. With the consumption or transformation of AS, the inhibitory effect gradually weakened. However, BM in AJ, especially *Acetobacter*, rapidly propagated and became the dominant bacteria under this condition. Then, a large amount of AA was metabolised continuously, which is a recognized as a good antibacterial substance. Therefore, AJ achieved high-efficiency and long-acting antifungal activity through this synergistic effect between AS and BM.

## Supplementary information

**Additional file 1.:** **Table S1.** Summary of qPCR primers. **Table S2.** Cycling protocols of qPCR. **Fig. S1.** IC_50_ of AJ against *B. cinerea*. Broken, horizontal and vertical lines represent the fitting line, half of the colony diameter of CK and the corresponding dosages, respectively. Black and red circles represent the observed value and the IC_50_, respectively. **Fig. S2.** Increase part of the inhibitory rate of AJ. Blue and green bar represents the inhibitory rate of AS(T2) and BM (T3) respectively. And the red bar represents the higher part of inhibitory rate of AJ compared to the AS and BM.

## Data Availability

All data and material generated or analysed during this study are included in this published article
