# Supplementary material for: A microbial ecosystem: agricultural Jiaosu achieves effective and lasting antifungal activity against Botrytis cinerea
Source: AMB Express. 2020 Dec 14;10:216. doi: 10.1186/s13568-020-01156-7 (PMC7736446; doi:10.1186/s13568-020-01156-7)
Supplement: Supplementary file 1 — Additional file 1.: Table S1. Summary of qPCR primers. Table S2. Cycling protocols of qPCR. Fig. S1. IC50 of AJ against B. cinerea. Broken, horizontal and vertical lines represent the fitting line, half of the colony diameter of CK and the corresponding dosages, respectively. Black and red circles represent the observed value and the IC50, respectively. Fig. S2. Increase part of the inhibitory rate of AJ. Blue and green bar represents the inhibitory rate of AS(T2) and BM (T3) respectively. And the red bar represents the higher part of inhibitory rate of AJ compared to the AS and BM. [file 13568_2020_1156_MOESM1_ESM.docx]

**Journal name** AMB Express

**Agricultural Jiaosu achieves effective and lasting antifungal activity against *Botrytis cinerea***

Yue Zhang^1^, Youhui Gao^1^, Zehui Zheng^1^, Xingyao Meng^2^, Yafan Cai^3^, Jianbin Liu^4^, Yuegao Hu^1^, Shuangdui Yan^5^ and Xiaofen Wang^1,*^

*^1^College of Agronomy and Biotechnology, China Agricultural University, Beijing 100193, China*

*^2^Beijing Technology and Business University, Beijing 100048, China*

^3^*Department of Biochemical Conversion,* *Deutsches Biomassforschungszentrum gemeinnütziges GmbH, Torgauer Straße 116, 04347 Leipzig, Germany*

^4^*Institute of Plant Nutrition and Resources, Beijing Academy of Agricultural and Forestry Sciences, Beijing 100097, China*

^5^*College of Resource and Environmental Science, Shanxi Agricultural University, Shanxi 030801, China*

***Corresponding author**. TEL.: +86 10 62733872; fax: +86 10 62731857

E-mail address: [wxiaofen@cau.edu.cn](mailto:wxiaofen@cau.edu.cn) (X. Wang)

**Table S1** Summary of qPCR primers

| Primer | Sequence | Target | Reference |
| --- | --- | --- | --- |
| F_alllact_IS | 5′-TGGATGCCTTGGCACTAGGA-3′ | *Lactobacillus* | Haarman et al. (2006) |
| R_alllact_IS | 5′-AAATCTCCGGATCAAAGCTTACTTAT-3′ |  |  |
| Ace-F | 5′-GCTGGCGGCATGCTTAACACAT-3′ | *Acetobacter* | Zhao et al. (2016) |
| Ace-R | 5′-GGAGGTGATCCAGCCGCAGGT-3′ |  |  |
| Bc-F | 5′-CAGGAAACACTTTTGGGGATA-3′ | *B. cinerea* | This study |
| Bc-R | 5′-GAGGGACAAGAAAATCGACTAA-3′ |  |  |

**Table S2** Cycling protocols of qPCR

| Target | Initial denaturation | Denaturation, annealing and extension | Cycles |
| --- | --- | --- | --- |
| *Lactobacillus* | 95°C, 5 min | 94°C, 15 s; 51°C, 2 s; 72°C, 50 s | 35 |
| *Acetobacter* | 95°C, 10 s | 95°C, 5 s; 56°C, 10 s; 72°C, 50 s | 40 |
| *B. cinerea* | 95°C, 2.5 min | 95°C, 15 s; 55°C, 20 s; 72°C, 50 s; 83°C, 15 s | 35 |


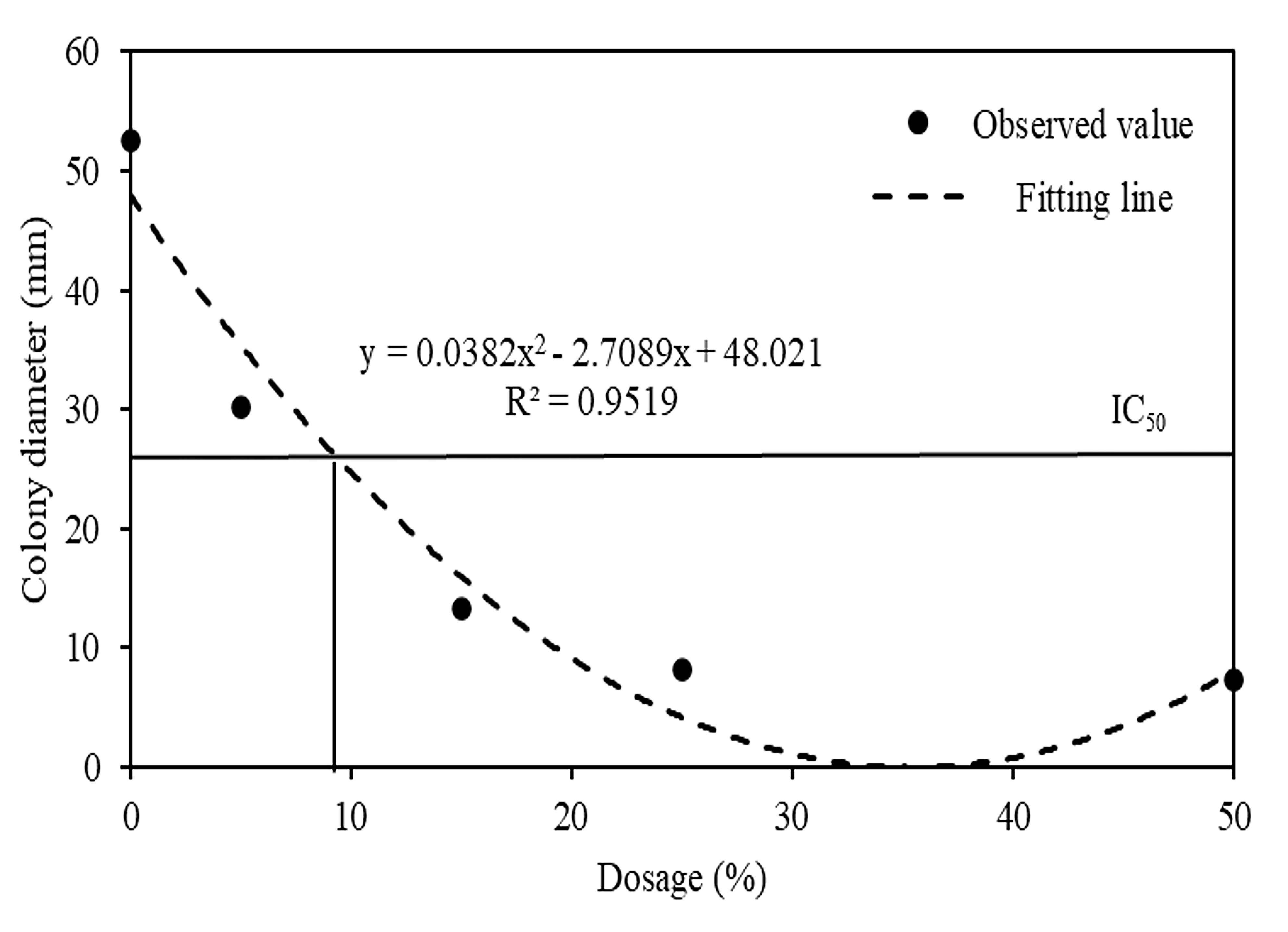


**Fig. S1** IC_50_ of AJ against *B. cinerea*. Broken, horizontal and vertical lines represent the fitting line, half of the colony diameter of CK and the corresponding dosages, respectively. Black and red circles represent the observed value and the IC_50_, respectively


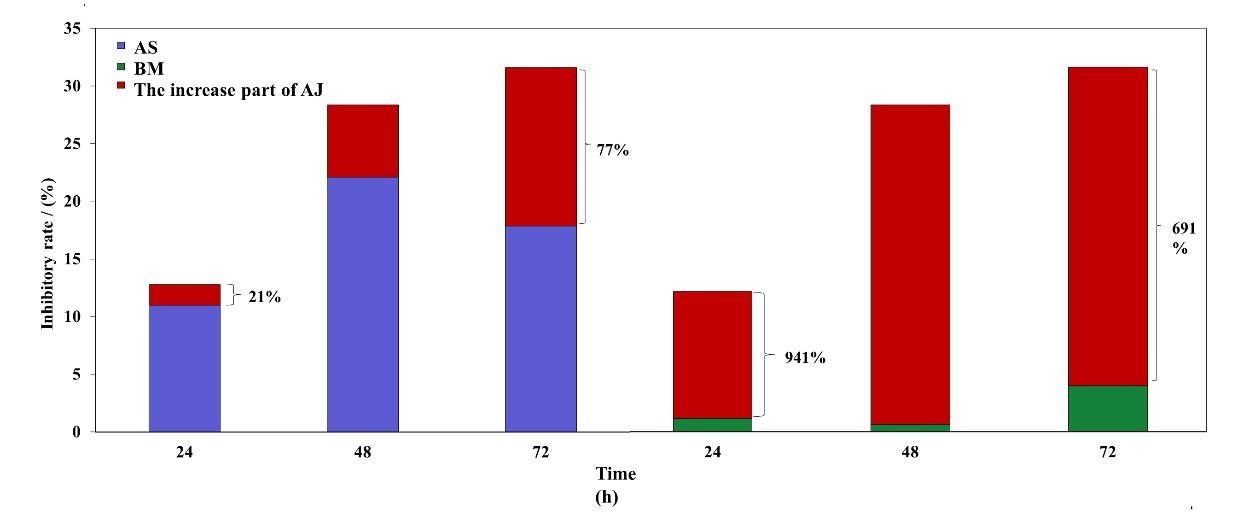


**Fig. S2** Increase part of the inhibitory rate of AJ. Blue and green bar represents the inhibitory rate of AS(T2) and BM (T3) respectively. And the red bar represents the higher part of inhibitory rate of AJ compared to the AS and BM.
